# The polysemy of psychotropic drugs: continuity and overlap between neuroenhancement, treatment, prevention, pain relief, and pleasure-seeking in a clinical setting

**DOI:** 10.1186/s12910-020-00497-z

**Published:** 2020-07-06

**Authors:** Eisuke Sakakibara

**Affiliations:** Department of Neuropsychiatry, The University of Tokyo Hospital, 7-3-1, Hongo, Bunkyo-ku, Tokyo, 113-8655 Japan

**Keywords:** Pain relief, Pleasure seeking, Neuroenhancement, Prevention, Psychotropic drugs

## Abstract

**Background:**

Enhancement involves the use of biomedical technologies to improve human capacities beyond therapeutic purposes. It has been well documented that enhancement is sometimes difficult to distinguish from treatment. As a subtype of enhancement, neuroenhancement aims to improve one’s cognitive or emotional capacities.

**Main body:**

This article proposes that the notion of neuroenhancement deserves special attention among enhancements in general, because apart from the notion of treatment, it also overlaps with other concepts such as prevention, pain relief, and pleasure seeking. Regarding prevention, patients’ mental endurance can be enhanced when a patient is prescribed a selective serotonin reuptake inhibitor for the purpose of preventing the relapse of depression following a stressful situation. As for pain relief, psychiatrists use medication to alleviate distress in patients who experience various types of anxiety; the alleviation of distress is equal to psychological pain relief, but is also an enhancement of the patient’s temperamental traits. Regarding pleasure seeking, insidious transition exists between neuroenhancement and pleasure seeking when using psychotropic drugs. It is well known that people use psychostimulants for recreational purposes and to induce overconfidence in one’s performance. The polysemy of psychotropics derives from their effects on human sensibility. Therefore, when using psychotropic agents, psychiatrists should pay close attention to what the agent is used for on each patient in each situation, and explicitly share the continuity and overlap in the purpose of prescribing a medication with the patients to make a better clinical decision.

**Conclusions:**

The notion of neuroenhancement overlaps not only with the notion of treatment, but also with other concepts of prevention, pain relief, and pleasure seeking. The continuity between those concepts makes the issues concerning the prescription of psychotropic drugs subtler. Psychiatrists should explicitly share the continuity with the patients to make a better clinical decision.

## Background

Improving human functions with the aid of biomedical technology beyond the purpose of treating disease or maintaining health is called enhancement [1]. Examples of physical enhancement include taking anabolic steroids for strengthening muscles and injecting erythropoietin for improving endurance. Among the various kinds of enhancement, neuroenhancement refers to improving mental functions utilizing biomedical technologies, such as psychotropic drugs and electromagnetic brain stimulation techniques [2, 3]. For instance, sharpening one’s memory and concentration using psychostimulants and turning a shy and nervous personality into an assertive and cheerful one with the aid of selective serotonin reuptake inhibitors (SSRIs) are typical examples of neuroenhancement [4, 5].

The moral status of enhancement has been highly debated in bioethics. Concerns regarding enhancement are not restricted to its uncertain safety and effectiveness. Among them, the concern about unfairness refers to the possibility that unequal distribution of biomedical resources employed for enhancement would result in further social inequality; the concern regarding coercion considers whether individuals would yield to direct or indirect demands from people surrounding them and unwillingly take enhancing medications; and the concern regarding complicity suggests the possibility that enhancement might strengthen arbitrary and sometimes unjust social values of the time [6, 7]. Finally, a more subtle but grave issue is that human authenticity might be lost by a dependence on biomedical technologies [8]. These concerns apply to the problem of enhancement in general.

Among biomedical technologies, this paper focuses on psychotropic medication in a psychiatric clinical setting. I maintain that neuroenhancement merits special attention and a separate discussion in the debate on enhancement in general, because the notion of neuroenhancement overlaps with multiple clinical concepts.

It is well acknowledged that enhancement is continuous with treatment, and the two are sometimes indistinguishable [7, 8]. For example, administration of growth hormone to those with short stature due to panhypopituitarism is considered treatment, while administering growth hormone to those with idiopathic short stature is considered enhancement, although the difference in purpose can be quite subtle [9]. The ambiguity between treatment and enhancement has profound implications for medical practice, since whether a certain medical intervention is categorized as treatment or enhancement often leads physicians to quite different conclusions.

**Fig. 1:**
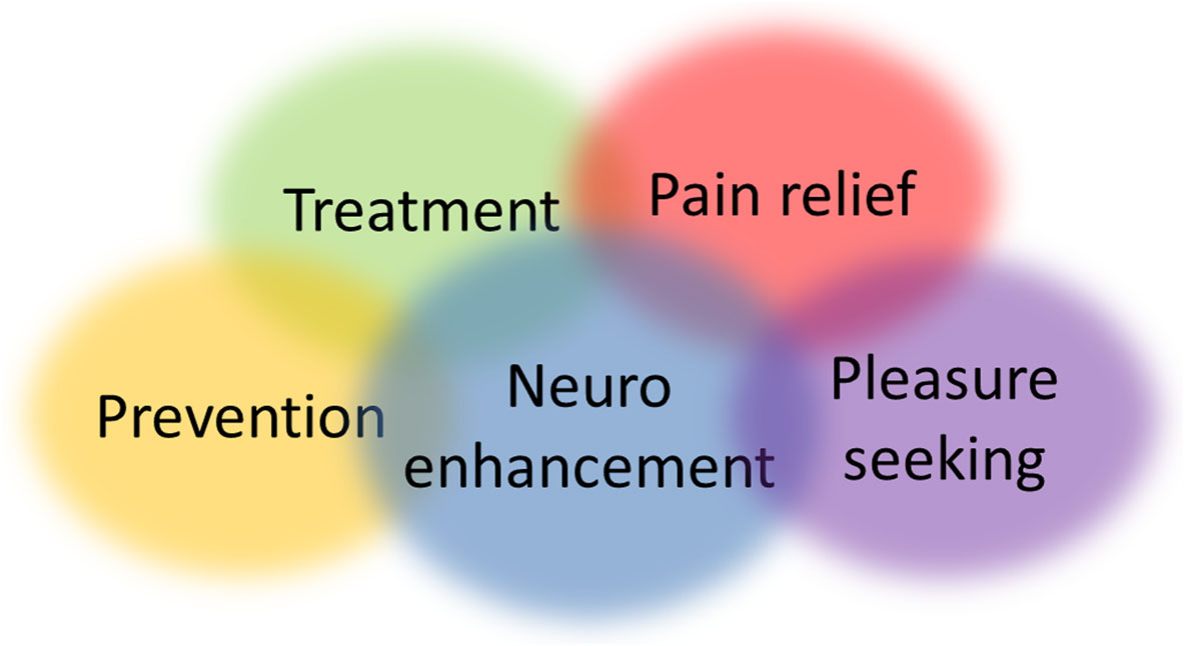
The overlap of concepts

In this paper, I will show that the use of psychotropic drugs for neuroenhancement is not only continuous with their use for treatment, but also continuous with their use for other purposes, including prevention, pain relief, and pleasure-seeking. The overlap of concepts is illustrated in Fig. 1. In the following sections, I will discuss each of these overlapping fields individually.

Although these purposes are continuous and overlap, each has a different moral implication. At the end of this paper, I point out that when prescribing psychotropic drugs, it is important to recognize what they mean to the patient at the time, and to share this information with the patient when making clinical decisions regarding pharmacotherapy.

## Main body

### Continuity of Treatment and Enhancement in Psychiatry

Enhancement is clearly distinguished from treatment when the drug user is simply aiming to achieve a high or enhance performance beyond normal levels. A typical picture of neuroenhancement is of healthy and ambitious students taking psychostimulants to improve their academic performance. It is sometimes difficult or even impossible, however, to distinguish enhancement from treatment of psychiatric illness when people have some psychological problem and begin to take psychotropic drugs to alleviate their hardship. This is because there is no established biomarker for psychiatric illness and the distinction between pathology and health is solely based on whether the alleged “symptoms,” such as anxiety or inattention, interfere significantly with one’s normal functioning or causes marked distress [10]. Based on these characteristics, a diagnostic system called the Hierarchical Taxonomy of Psychopathology (HiTOP) has been proposed that describes mental disorders as comprising multiple dimensions and assumes a continuum between normality and pathology [11].

Attention for the problem of neuroenhancement is growing as the numbers of SSRI and psychostimulant prescriptions increase from those in the 1990s [12]. A part of this increase in prescriptions is arguably attributable to the fact that drugs have finally reached individuals with obvious psychiatric illness who were formerly untreated with psychotropic medications. However, it must also be a consequence of the proliferation of prescriptions for those who have never been considered as having psychiatric illness nor have required medication for its treatment [13, 14].

Psychiatric illness has been gradually expanding even though the concept of illness and diagnostic criteria are roughly unchanged. McNally describes the phenomenon as “bracket creep,” in which citizens living in a country with progressive taxations become obliged to pay higher and higher taxes because of the rise in prices and nominal wages despite unchanging tax rates [15]. In other words, this is the process of medicalization, whereby those who have not been categorized as pathological are gradually incorporated into the object of medicine. In a sense, neuroenhancement is spreading in the name of treating illness.

In addition, there are cases in which enhancement and treatment are conceptually indistinguishable in psychiatry. The notion of treatment has the connotation of reinstating the patient to his/her premorbid status. However, from the viewpoint of a mechanism of action, psychotropic drugs do not restore the patient’s premorbid physiological state. As is well known, SSRIs are effective for treating depression, but not because depression is caused by a serotonin deficiency [16]. Psychotropic drugs act therapeutically by establishing a new equilibrium in the brain that is different from the premorbid status.

Among various psychiatric disorders, those with clear onset and subacute clinical course, such as depression and schizophrenia can conform to the medical model. For those illnesses, remission of symptoms and recovery of the premorbid health status are, though not always achievable, a prima facie goal of treatment. However, contemporary psychiatrists face other psychiatric disorders for which what constitutes as a remission of symptoms is unclear.

For example, SSRIs are prescribed for anxiety disorders; however, some patients with anxiety disorders have anxious temperament and lifelong neuroticism, which are primarily a part of their personality. Additionally, psychostimulants are prescribed for those with attention deficit/hyperactivity disorder (ADHD). ADHD is a neurodevelopmental disorder – a disorder of native neurocognitive function. These conditions have neither a clear time of onset nor “premorbid” levels of functioning with which we can define “remission of symptoms” and “recovery from illness.”

In contemporary psychiatry, psychotropic drugs are also prescribed for those conditions in the name of “treatment of illness.” Yet the “treatment” here is nothing but the improvement of an individual’s natural mental capacities to reach a level of functioning expected by society. Therefore, aside from the level of functioning before the initiation of psychotropic drugs, this “treatment” is not different from neuroenhancement.

Because treatment and enhancement are continuous, concerns raised regarding enhancement are also relevant for pharmacotherapy used to treat psychiatric disorders, as far as borderline cases are concerned. Psychiatrists have a particular regard for safety and efficacy of pharmacological treatment. In contrast, issues regarding coercion and complicity are blind spots for healthcare professionals: once a condition is categorized as an illness, it is considered objectively and universally harmful, and that it should be removed without question. Healthcare professionals are prone to overlook the possibility that recommending pharmacotherapy might be perceived as coercion for the “identified” patient or complicity with the local society’s values.

The possibility of complicity is concrete. For example, the prevalence of social anxiety disorder is 6.8% in the United States, whereas it is 0.7% in Japan [17, 18]. One explanation of this difference is underestimation of prevalence in Japan due to stigma against mental illness. However, another interpretation is that social anxiety is more pervasively pathologized in individualistic societies like the United States than in collectivistic societies like Japan [19]. This second interpretation is supported by a study finding that university students in collectivist societies have higher levels of social anxiety and embarrassment but are more likely to accept socially reticent and withdrawn behaviors [20]. Regarding the latter interpretation, the diagnosis and prescription of SSRIs for social anxiety disorder in the United States runs the risk of complicity with US culture, in which assertiveness is highly valued.

Furthermore, it has been reported that the prevalence of ADHD is increasing around the world and varies across countries [21]. In the United States, the percentage of adolescents who are medicated with stimulants under a diagnosis of ADHD is particularly high among affluent white communities, which may reflect the stringency of performance norms in the respective societies.

### Continuity between Treatment, Prevention, and Enhancement

Vaccination and water fluoridation are typical examples of utilizing biomedical technology for preventing disease. The moral status of prevention is different from that of enhancement, and is rather akin to that of treatment [7]. When biomedical technology is utilized for prevention of disease, safety/efficacy of the intervention becomes the point, while ethical concerns about coercion, complicity, and authenticity are not usually propounded. Similar to treatment, the problem of fairness with respect to prevention is usually addressed by granting citizens subsidies or providing occasions to receive the benefit of prevention for free.

Recently, interventions aimed at disease prevention tend to be performed within the context of treatment. For example, lifestyle diseases, such as essential hypertension, dyslipidemia, and osteoporosis are currently regarded as diseases, and physicians commonly treat them with pharmacotherapy [22]. In contrast, some researchers maintain that these conditions are not diseases themselves, but the risk factors for disease, and that pharmacological interventions for those conditions should be categorized as prevention [23].

The situation becomes more complicated because prevention and enhancement are sometimes not clearly distinguishable in psychiatry. For instance, the presence of sub-threshold depressive symptoms that do not satisfy the diagnostic criteria of major depression is a risk factor for later development of diagnosable major depression [24]. Therefore, interventions for alleviating sub-threshold depressive symptoms could be considered as (primary) preventive measure for major depression. However, depressive symptoms, even if they are sub-threshold, are different from physical conditions like hypertension and osteoporosis in that the former accompanies unpleasant experiences and decrease in functioning. Alleviating sub-threshold symptoms improves one’s mood and functioning. Therefore, introducing pharmacotherapy for sub-threshold symptoms can be categorized not only as prevention, but also as a kind of neuroenhancement.

A more subtle case is continuing long-term maintenance pharmacotherapy for those who have remitted major depression. Antidepressant medications should be continued for a certain period (usually 6 months or so) after the remission of symptoms for relapse prevention [25]. Thus, what should be done if the living environment of the patient has drastically changed and he or she begins to face a stressful environment just when the physician tries to terminate antidepressant medications? In this situation, it is reasonable to postpone the discontinuation of antidepressants for a while to prevent the recurrence of depression. However, prolongation of maintenance pharmacotherapy is sometimes difficult to distinguish from the enhancement of psychological endurance to survive stressful environments; when the stress faced by the patient is minor enough to be tolerated by a person of average endurance, then the intervention can safely be called prevention. However, as the burden from the environment increases, the medication to make it tolerable will take on increasing tints of enhancement.

### Continuity between Treatment, Pain Relief, and Enhancement

The concept of pain relief interlaces with the concept of treatment in psychiatry in multiple respects.

First, psychiatric disorders often accompany pain problems. For example, major depression is sometimes accompanied by pain, such as headache, which is resolved as the depression remits [26].

Second, one influential theory of chronic pain maintains that the development and maintenance of chronic pain is mediated by “fear of pain” and excessive avoidance of circumstances that one conceives might induce pain [27]. In this theory, what is called “negative affectivity” is thought to aggravate the fear of pain and excessive avoidance. Negative affectivity is a psychological trait associated with the frequent, intense experience of negative emotions [28]. Therefore, it is possible that negative affectivity, as defined here, might be conceptualized as a mood disorder or anxiety disorder, and become a target of pharmacological treatment in psychiatry.

Third, the association between ADHD and fibromyalgia, a syndrome characterized by chronic widespread musculoskeletal pain, has long been suspected [29]. For ADHD patients reporting chronic pain, psychostimulants may be beneficial not only for alleviating neurocognitive symptoms, but also for relieving their chronic pain [30].

Fourth, although tricyclic antidepressants and serotonin and norepinephrine reuptake inhibitors were first developed for the treatment of depression, they are now also frequently utilized for the treatment of chronic pain [31].

In the case of pain that accompanies physical disorders, such as cancer pain, pain relief and treatment of the underlying illness are conceptually distinguishable, such that pain relief is clearly categorized as a symptomatic therapy. In contrast, psychiatric disorders are defined by an ostensible disturbance in behavior and subjective experiences, and alleviating the disturbance is an essential part of the treatment of psychiatric illness. Those who seek psychiatrists usually feel emotional distress. The mission of psychiatry is, directly or indirectly, linked to removing or mitigating the distress patients suffer. Bearing in mind that mitigating the distress is equivalent to relieving psychological pain, the boundary between treatment and pain relief is more ambiguous in the realm of psychiatry than in the realm of internal medicine.

The distinction between enhancement and pain relief is also ambiguous. Pain is sometimes derived from actual stressors or overload in daily living. For example, it is known that elite athletes consume more pain killers than the general population [32]. Athletes use larger amounts of pain killers because they are likely to hurt themselves through intensive training, and because they prioritize winning a match and achieving higher scores before resting, even when they experience pain. Also relevant are the influence of the culture of elite athletes, where yielding to pain is considered soft and “unmanly,” and the pressure from teammates that requires individual athletes to devote themselves to the victory of the team at any time [32]. Similar to enhancement, pain relief elicits concerns about complicity and coercion.

Comparable situations to an athlete’s heavy use of pain relievers are observed in the realm of psychiatry. For example, relying on psychotropic drugs to relieve distress, rather than decreasing psychosocial stressors by changing situations that elicit interpersonal and/or occupational hardship can be interpreted as enhancement of endurance against psychological stress. However, some researchers have made critical remarks about this behavior, calling it a “pharmacological Band-Aid” [15] and “Aspirin for the mind” [33], because it is only numbing the sensitivity to distress rather than removing the cause of distress.

In addition, employees and students who admitted to having used psychotropic drugs to improve cognitive functioning or elevate their mood are likely to have mental disorders, suffer from frequent or long-term stress, and use illicit drugs, rather than being healthy and ambitious people who aim to become “better than well” [34]. This would provide further evidence that neuroenhancement is continuous with self-medication for therapeutic and pain-relieving purposes.

The use of psychotropic drugs at the intersection of pain relief and enhancement is problematic because pain is an alarm from one’s body; continuing to neglect the alarm of pain, although enabling higher performance in the short term, endangers the long-term health of the individual.

### Continuity between Pain Relief, Pleasure Seeking, and Enhancement

In the United States, the overuse of opioids has become a subject of public concern [35].

In 2017, 47,000 people died from opioid overdose in the country [36]. It has been argued that the “opioid crisis” was caused by pharmaceutical companies’ clever promotion of new opioid products, in addition to the closer monitoring and control of pain as “the fifth vital sign” for advancing patient-centered care [37]. The transition from appropriate use of opioids for pain relief into opioid dependence is mediated by using opioids for “chemical coping,” in which pain relievers are used for alleviating psychosocial distress rather than physical pain [38].

Transition also occurs between the use of psychotropic drugs for neuroenhancement and pleasure seeking. People long for the enhancement of their own capacity because they believe that it facilitates their pursuit of happiness. Yet, among what is called the “pursuit of happiness,” achieving true prosperity (eudaimonia) and indulging in subjective pleasure (hedonia) is quite different [39]. Yet, it can be difficult for one to discern whether the use of a psychotropic drug is facilitating *eudaimonia* or only misleading him/her into indulging in its *hedonic* properties because psychotropic drugs affect human sensibility.

For example, it is well known that psychostimulants are frequently abused for recreational purposes, although the development of extended-release formulations has decreased the risk of abuse significantly [40]. In one study, among university students who affirmed the off-label use of prescription psychostimulants for ADHD, 54% reported using psychostimulants exclusively for academic reasons, whereas 40% admitted that they used it both for academic and nonacademic reasons, including “to get high” and “to feel better” [41].

In contrast to physical enhancement, such as muscular strengthening and stature lengthening, it is often not easy to verify the specific effects of neuroenhancement. Therefore, even if the use of psychotropic drugs was initially aimed at neuroenhancement and transformed into pleasure seeking, psychotropic drug users can deceive themselves and falsely believe that they are using drugs to improve their mental capacities. This concern takes on reality when we consider the previous finding that psychostimulants not only enhance cognitive performance, but also induce overconfidence in one’s own performance [5]. A recent study indicated that approximately 65% of college students who use stimulants believe them to be effective in improving their academic performance [42]. However, the GPA of college students who had never used stimulants increased as the school year proceeded, whereas the grades of the students who started or continued to use stimulants did not improve [43].

### Overlap of Concepts: Causes and Implications

The moral status of psychotropic drug use is more complicated than that of a chemical agent for physical enhancement because the effects of psychotropic drugs are polysemous, which derives from the fact that they affect human sensibility. The way we feel sometimes constitutes psychiatric illness. The things we feel, such as pain and pleasure, are often a subjective reflection of actual hardship or prosperity. The way we feel may bring about a change in reality by affecting our behavior. Therefore, drugs that act on one’s sensibility are sometimes regarded as remedies for psychiatric disorders, but in other cases may be considered agents that affect subjective feelings while leaving the actual problem untreated. In other cases, they are thought to change reality via the transformation of subjectivity.

Psychotropic drugs are chemical substances, with names such as “antidepressants” or “pain relievers,” that are relative to human interests. Therefore, it is possible that the same substance acts as a remedy for one person, while it is a neuroenhancing agent for another. Additionally, the same substance may assume different meanings for different occasions, even within the same person. For example, it is possible that a substance formerly taken for neuroenhancement turns into an object of addiction without one’s knowledge or intention.

The overlap of five concepts—treatment, neuroenhancement, prevention, pain relief, and pleasure-seeking—has considerable clinical implications. When prescribing psychotropic drugs, clinicians should pay close attention to what they are to be used for by each patient in each situation. That is because each of these concepts carries distinct moral entailments.

“Treatment” is a kind of intervention that is medically necessary and is a realm in which health care providers must be engaged. Interventions to treat illness are often urgent and a more adventurous use of drugs may be tolerated. In some cases, treatment without or against patients’ consent may be justified on the basis of the principle of benevolence.

In contrast, interventions to prevent illness are not urgent, and there is a problem of “false negatives” that can lead to unnecessary interventions. Therefore, they must be based on robust evidence that the benefit of the intervention exceeds its risk [44]. In addition, consideration must be given to the possibility that it will stigmatize the individuals to whom preventative measures are administered [45]. Another problem is that once preventative interventions are started, it is difficult to determine how long they should be continued.

“Enhancement” is continuous with treatment in that they both promote an individual’s welfare [46]. However, unlike treatment, enhancement falls outside the scope of medical necessity and is not covered by health insurance under current social conventions. Furthermore, as the goal of augmenting a function is less likely to be shared by people in general than the goal of curing an illness, enhancement is more likely to invite concerns over coercion and complicity. There is a convincing opinion that enhancement should be based on one’s free will and that it should not be applied to nonautonomous individuals, such as minors, to protect their open future [47].

“Pain relief” is directly related to people’s quality of life. However, it has long been downplayed by medicine, which has recognized the treatment of illness as its primary role. Pain relief is essential to achieve patient-centered care, and physicians should be actively involved in it. It must also be kept in mind that although pain relief is of great benefit to patients in the short term, it may be harmful in the long run [48].

Finally, “pleasure seeking” is a common motive of daily activities outside of medicine in which medicine is strongly discouraged from involvement. It is up to the individual to decide whether to pursue pleasure-seeking within the limits set by law, but it may be detrimental to her prosperity. In addition, the pursuit of pleasure runs the risk of falling into addiction. A physician who notices that a person is indulging in psychotropic drugs for recreational purposes that are detrimental to her welfare is expected to give her appropriate advice and support her in treating her addiction.

These five concepts are not mutually exclusive. That is, a given prescribing case may not always fall exactly into one category. Instead, it is often more aptly described as being located somewhere on a spectrum between two or three concepts, e.g., between treatment and enhancement, and between enhancement and pain relief.

Despite the overlapping and continuous nature of the concepts, clinical decisions must be made in an *all-or-nothing* manner. In other words, a medication *is* or *is not* prescribed. Therefore, based on the idea of shared decision making, psychiatrists should explicitly share ambiguities in the purpose of prescribing a medication with patients to make a better clinical decision [49]. In the face of the polysemy of psychotropic drugs, “ethically informed, ecologically sensitive clinical practices” are needed [21].

## Conclusions

Regarding physical enhancement, it has been long pointed out that the borderline between treatment and enhancement is ambiguous. In this paper, it is shown that the notion of neuroenhancement is not only continuous with the notion of treatment, but also with that of prevention, pain relief, and pleasure seeking. The ambiguity of its purpose is derived from the fact that psychotropic drugs affect human sensibility. Therefore, when using psychotropic agents, psychiatrists should pay close attention to what they are used for on each patient in each situation, and explicitly share the ambiguity and overlap in the purpose of prescribing a medication with the patients to make a better clinical decision.

## Data Availability

Not applicable.

## Abbreviations

ADHD: Attention deficit/hyperactivity disorder
SSRI: Selective serotonin reuptake inhibitor
HiTOP: The Hierarchical Taxonomy of Psychopathology
